# Tooth-to-white spot lesion YOLO: a novel model for white spot lesion detection

**DOI:** 10.1186/s12903-025-06936-w

**Published:** 2025-10-09

**Authors:** Hau Man Chung, Jingjing Ke, Mengdan Zhang, Lixian Kong, Junming Zheng, Lusai Xiang

**Affiliations:** 1 Guanghua School of Stomatology, Hospital of Stomatology, Sun Yat-Sen University, Guangdong Provincial Key Laboratory of Stomatology, No. 56 Lingyuan West Road, Guangzhou, Guangdong 510055 China; 2 Foshan Stomatological Hospital, School of Stomatology and Medicine, Foshan University, No. 5, Hebin Road, Chancheng District, Foshan, Guangdong 528000 China

**Keywords:** White spot lesions, Object detection, Deep learning, Explainability analysis

## Abstract

**Background:**

To develop a new deep learning model for detecting white spot lesions (WSLs), which are commonly observed in patients undergoing orthodontic treatment, and assess its accuracy.

**Methods:**

A total of 653 intra-oral photographs of WSLs were collected and annotated. Our novel model, tooth-to-WSL You Only Look Once (TW-YOLO), and the original YOLOv5 model were fine-tuned and evaluated, with 457 photographs used for training; 130, for validation; and 66, for hold-out testing. Cohen's kappa coefficient between model prediction and orthodontist annotation was used as the primary evaluation metric, and mean average precision (mAP@0.5:0.95), average precision (mAP@0.5) and F1 score were also evaluated. The score-CAM technique was used for explainability analysis.

**Results:**

Cohen's kappa coefficient values were 0.76 and 0.62 for TW-YOLO and YOLOv5, respectively. The mAP@0.5 and mAP@0.5:0.95 were 0.78, 0.51 for TW-YOLO and 0.69, 0.45 for YOLOv5, respectively. Explainability analysis suggested that the TW-YOLO model could implicitly learn the distribution pattern of WSLs by shifting more attention toward these regions.

**Conclusion:**

Compared to original YOLO model, our novel TW-YOLO model demonstrated improved accuracy. Smaller proportion of small sized object and examine tooth enamel at original resolution contributed to this improvement.

**Supplementary Information:**

The online version contains supplementary material available at 10.1186/s12903-025-06936-w.

## Introduction

The stage before cavitation in the development of dental caries is called white spot lesion (WSL). It is characterized by subsurface demineralization areas formed under an intact enamel surface. WSLs Manifest as alterations of the translucent feature of the enamel, and the color of these areas appears opaque white. The reported incidence of WSLs is widely variable, but on average, such decalcifications are found in 30–70% of patients during orthodontic treatment [1]. The high incidence of WSLs necessitates attention from patients and practitioners. Early detection of WSLs during orthodontic treatment would allow the implementation of preventive measures and control of the demineralization process before it further progresses [2]. Various techniques are used to detect WSLs, including visual inspection, photography, light-induced fluorescence, and quantitative laser analysis. The enamel decalcification index (EDI), a visual inspection tool designed to categorize and assess the presence and severity of enamel defects [3], not only has an accuracy comparable to that of light-induced fluorescence [4] but also is easier to implement and can sometimes be used for patient self-monitoring, which is helpful for early detection [5]. However, using the EDI requires professional training, which limits its applicability in patient self-monitoring. This method is also time-consuming, which makes it impractical for large-scale screening and dynamic monitoring during orthodontic processes.

Recent advances in the field of deep learning have shown promising potential for streamlining the routine work of dental caregivers and empowering patient self-monitoring [6, 7]. Many studies have evaluated the classification ability (the ability to differentiate between images of teeth with and without lesions) of deep learning models. Askar et al. demonstrated that SqueezeNet, a convolutional neural network, performs this task with great accuracy [8]. Zeng et al. developed a multi-stage deep learning-based pipeline of artificial intelligence algorithms that is capable of assigning ICDAS score for each tooth, and demonstrated 89.78% sensitivity and 91.67% specificity for white spot lesions [9]. Determining the size and location of these lesions, which usually involves object detection and semantic segmentation, is the next step. This is especially relevant in the prevention and management of WSLs because location and size influence esthetic outcomes.

Studies indicate that deep learning models can localize and identify caries in bitewing X-rays with a recall of 0.727 and an F1 score of 0.687 [10, 11]. Furthermore, Casalegno et al. used a U-Net–like network architecture for caries segmentation in near-infrared transillumination (TI) images, achieving a mean intersection over union (mIOU) of 72.7% and an area under the ROC curve (AUC) of 85.6% [12]. Wang et al. further incorporated sub-band fluorescence image, and demonstrated an accuracy of 96.4% [13]. These findings collectively demonstrate that deep learning models exhibit not only high processing speed but also substantial diagnostic accuracy in caries detection.

Compared with radiographs and fluorescence imaging, intra-oral photographs are more readily accessible and often used in the evaluation of WSLs. But applying deep learning tools in the reading of intra-oral photographs poses its own challenges. Compared with studies analyzing dental radiographs and fluorescence images, in which the lesions comprise the majority of the image, intra-oral photographs are usually much larger, and the WSLs comprise a much smaller proportion. Some researcher choose to crop images manually first then use them for training and prediction. In study done by Zang et al., they perform both training and prediction of caries lesions in intra-oral images that only include the tooth with caries and adjacent ones [14]. Similar preprocessing was performed by Li et al. to detect dental caries on images containing only a single tooth [15]. However, in some other studies [16, 17], intra-oral photographs are first significantly downscaled before being fed into deep learning networks. Although these studies have also achieved relatively good detection accuracy through the adoption of more advanced network architectures, the data loss caused by image size reduction has inevitably affected their ability to achieve higher accuracy. As demonstrated in the study by Ozsunkar et al., a relatively poor accuracy result for WSLs detection was obtained by directly applying YOLOv5 in an end-to-end approach [17]. Therefore, instead of simply downsizing an intra-oral photograph, a novel deep learning model is needed to fully utilize rich texture data in the original image. Inspired by the task partitioning paradigm [18] and the sliding windows strategy [19, 20], we developed a tooth-to-WSL You Only Look Once (TW-YOLO) model and compared its accuracy metrics with those of YOLO. The hypothesis to be tested is whether there are difference in accuracy between TW-YOLO and YOLO. We also implemented explainability analysis to gain a better understanding of the mechanisms behind model decision-making. To the best of our knowledge, our study is the first to implement explainability analysis on object detection models within the domain of dental medicine.

## Materials and methods

### Data collection

The current retrospective study acquired anonymized intra-oral photographs of orthodontic patients with WSLs from image archives in the Orthodontics Department of Foshan Stomatological Hospital, Foshan University. All intraoral frontal view photographs taken from January 1 st, 2020, to October 31 st, 2024, were included for further selection. Two senior orthodontic specialists assessed whether WSLs were present in the photograph, where WSLs were defined as ICDAS scores of 1 to 2. In case of disagreement, the decision was made by principal investigator. Only photographs with WSLs were included in the database. Patient de-identification was done by removing all meta data from image file, and covering the patient's nose and eyes in the photograph. The protocol of the current study was approved by the Ethics Committee of the Foshan Stomatological Hospital, Foshan University (2024-FSKQ-LW-002). All patients signed informed consent at the beginning of their treatment sessions to have their anonymized image data to be used for medical research purpose. In total, 653 anonymized intra-oral pictures were collected, a number greater than those in previous studies [8, 21, 22].

All intra-oral photographs were taken from patients receiving fixed appliances orthodontic treatment, either before the treatment commenced or after appliance removal, with a digital reflex camera (Canon EOS 60D, Canon Corp., Tokyo, Japan). All images were taken from frontal view. The image resolution was approximately 4000 by 3000 pixels. Tooth surfaces were cleaned and dried prior to intra-oral photography.

### Image annotation and data augmentation

All image data were first annotated manually by two senior orthodontic specialists with LabelImg [23] by drawing bounding boxes around WSLs. The specialists first annotated 10 cases together, reaching a consensus, and then each specialist continued the annotation work independently. Finally, only regions selected by both orthodontic specialists were kept as annotations. Bounding boxes around individual teeth were drawn by a junior researcher. The schematic for annotation process is shown in Supplementary Fig. 1.

**Fig. 1:**
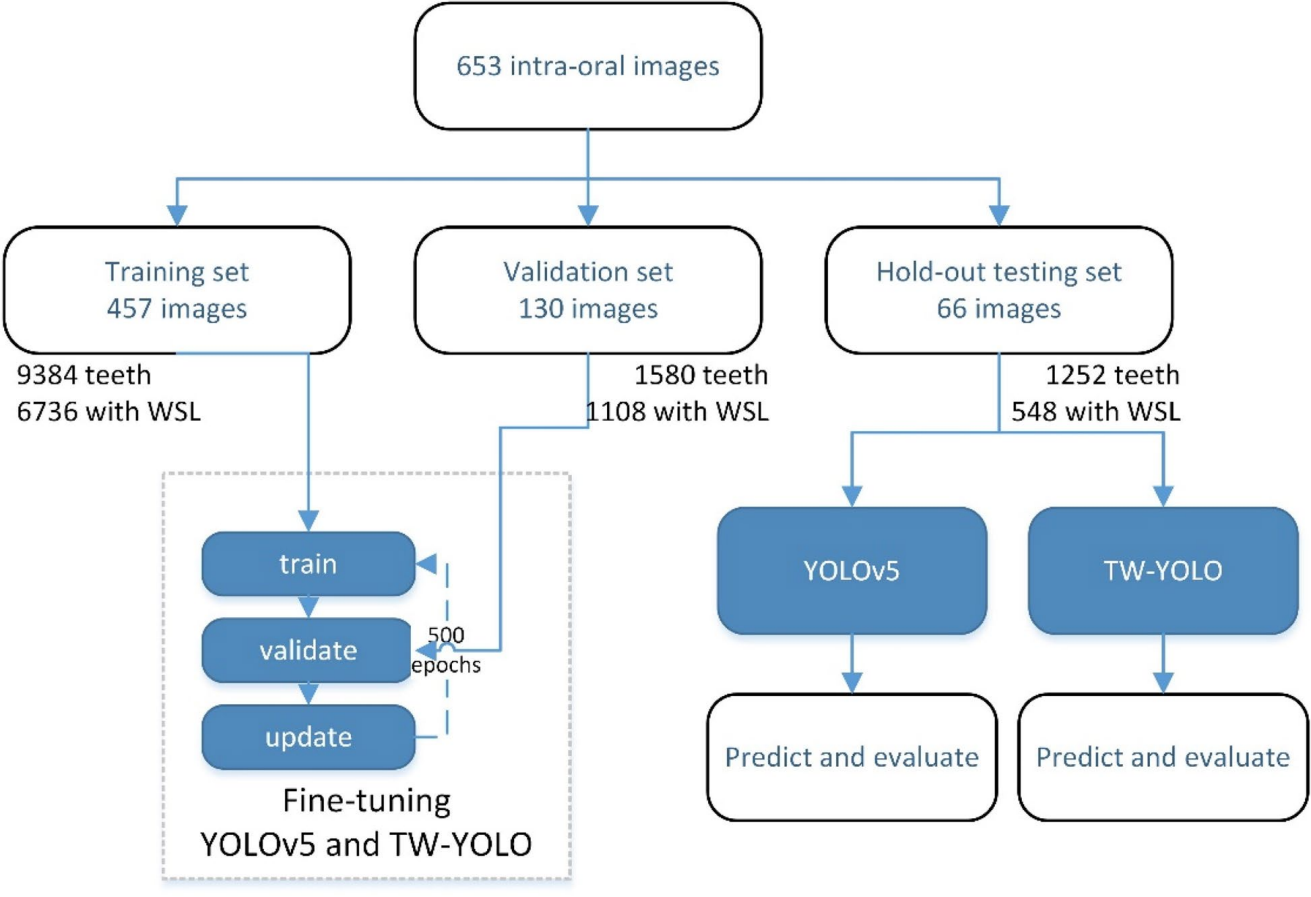
Workflow of model finetuning and evaluation

With a previous study [24] used as a guide, the 653 intra-oral photographs were randomly split into three groups (Fig. 1): 457 images were used for training; 130, for validation; and 66, for hold-out testing. The images in the train-validation dataset (90% of all images) were used for model training and validation, and the images in the hold-out testing dataset (10% of all images) were used for performance evaluation. The images in the hold-out testing dataset were never seen by the deep learning models, to avoid memory effect in model training [25].

Data augmentation techniques, including random cropping and scaling, image rotation, and affine transformations, were employed to expand the effective dataset scale and enhance the spatial robustness of the deep learning model.

The minimum sample size was estimated based on the approach outlined in a previous study [26]. Based on a pilot study, we estimated that Cohen's kappa coefficient values were 0.6 and 0.77 for YOLOv5 and TW-YOLO, respectively. On average, WSLs covered 15% of the tooth enamel surfaces in the photographs. Given these parameters, at least 505 teeth with WSLs were needed. The external testing dataset included 548 teeth with WSLs, satisfying the minimum sample size requirement.

### TW-YOLO model architecture

**Fig. 2:**
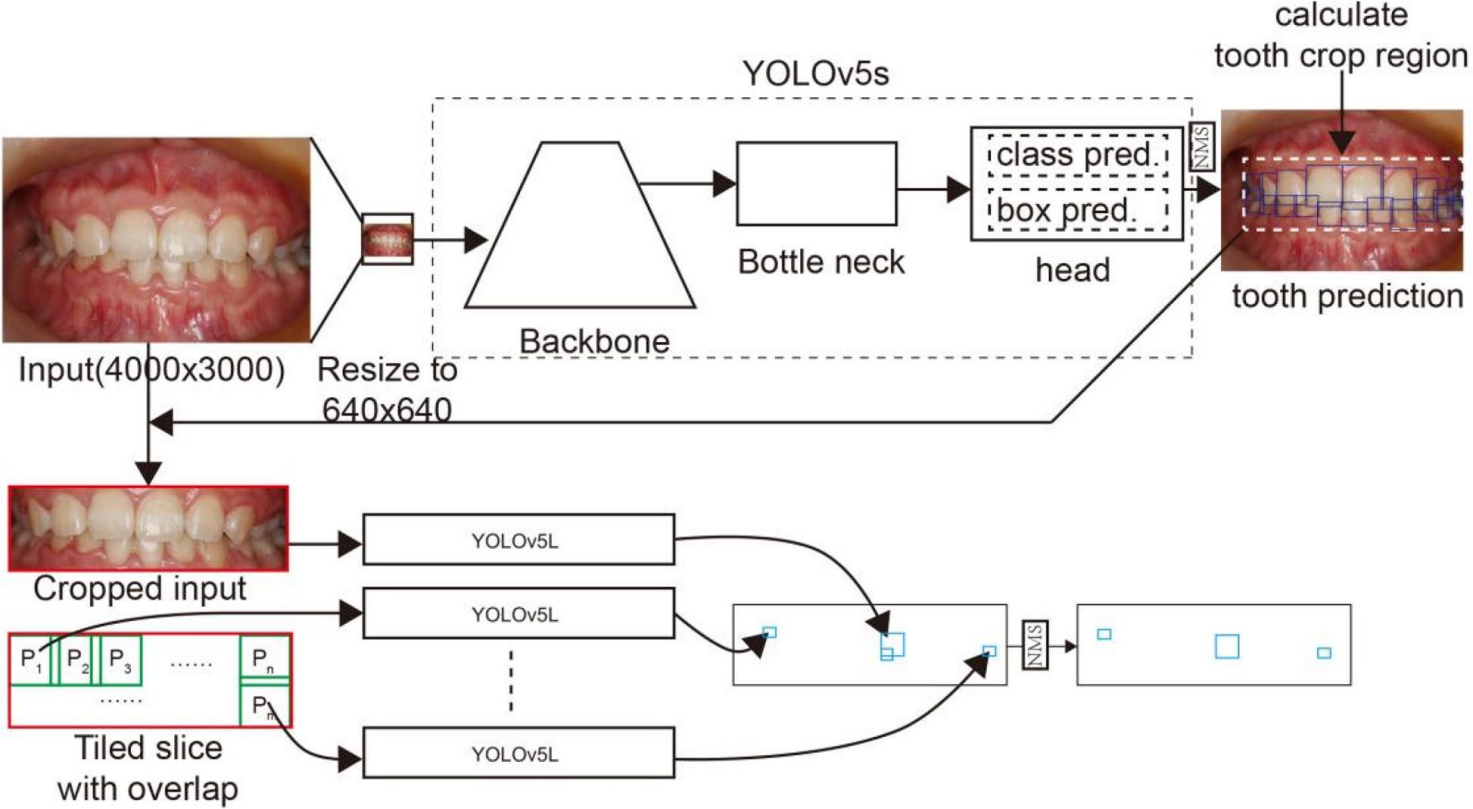
Scheme chart of the tooth-to-WSL YOLO model

To mitigate the issue of excessive downsizing in conventional image preprocessing of high-resolution intra-oral photographs, we developed the novel TW-YOLO model (Fig. 2). First, intra-oral photographs (approximately 4000 × 3000 pixels) undergo standard proportional resizing to fit within a 640 × 640 pixels square. The resized images are then processed by a YOLOv5s network to localize teeth. Non-maximum suppression (NMS) is subsequently applied to retain only non-overlapping predicted bounding boxes. Based on the bounding boxes for the teeth, the image region encompassing all detected teeth is calculated. This region is then cropped out of the original, full-resolution image. Within this cropped input, tiled image extraction is performed using sliding 640 × 640 pixels windows. Adjacent windows overlap by 50 pixels to prevent lesion oversight.

Next, all extracted image tiles along with the entire cropped input are processed individually by the YOLOv5l network to detect WSLs. This facilitates concurrent localized detection within the tiles and holistic detection on the cropped input. During the training phase, each extracted tile is treated as an independent training sample. For the prediction phase, the detection results from all processed tiles and the cropped input are mapped back onto the coordinate space of the original, uncropped image. A final NMS step is applied to eliminate significantly overlapping bounding boxes, retaining the post-processed detections.

### Fine-tuning of YOLO and our TW-YOLO model

YOLO models possess parameter counts in the millions (YOLOv5s: 7.2 million; YOLOv5l: 46.5 million), so training such models from scratch is impractical. Therefore, we initialized both models using pretrained weights (trained on the common objects in context [COCO] dataset) as starting points for fine-tuning. This transfer learning strategy leveraged the generic feature extraction capabilities acquired from broad image data while incorporating domain-specific knowledge pertinent to orthodontic WSL detection.

Fine-tuning of the YOLO network was performed by training models for 500 epochs with adaptive optimization hyperparameters. Specifically, batch size was set to 4 images per batch, learning rate initially was set to 0.01, final learning rate was set to 0.0001, Stochastic Gradient Descent (SGD) was used as optimizer. The loss function, comprising object detection loss (computed via intersection over union [IOU]) and classification loss (computed via Cross-Entropy), was evaluated in each epoch and backpropagated to update model weights. Pytorch framework (v2.8, on CUDA 12.6, python 3.12) was used for model finetuning and evaluation.

### Model performance evaluation

After training, model performance was evaluated using the testing dataset. The primary evaluation metric was the pixel-wise Cohen’s kappa coefficient. We adopted this metric to evaluate the agreement between our orthodontists and the models regarding the boundaries of the WSLs. Other accuracy metrics included overall mean average precision (mAP@0.5:0.95), which was first introduced by the COCO detection challenge and has since become the most common evaluation metric for object detection accuracy. Average precision at the 0.5 IOU threshold (AP@0.5) and F1 score were chosen as secondary accuracy metrics. The evaluation metrics, including IOU, precision (P), recall (R), mAP@0.5:0.95, mAP@0.5, and F1 score, were calculated using methods outlined in prior research [8, 27]. The prediction time for each image was also tracked and compared.

We would like to point out that for object detection task, a true negative means no detection is overlapped with background, and it is not applicable. Thus, both ROC curve and the area under curve (AUC) can’t be calculated. On the other hand, average precision, whether the mAP@0.5:0.95 or mAP@0.5, is the area under precision recall curve by definition. A more detailed description of the calculation algorithms for metrics in our study is included in the Supplementary Information.

### Explainability analysis with ablation-CAM

Gradient-weighted class activation mapping (grad-CAM) uses the gradients of any target concept flowing into the final convolutional layer to produce a coarse localization map that highlights important regions in the image for predicting the concept [28]. It is widely used in explainability analysis of classification problems [8, 29, 30]. However, for object detection problems, both class discrimination and target localization are equally important. While the prediction score can be used for classification explainability, to better understand how deep learning models draw bounding boxes, we investigated the IOU ratio between the predictions from the models and the annotations from the orthodontic specialists. To calculate gradients for this purpose, however, is a tricky task. To solve this problem, Wang [31] proposed score-based class activation mapping (score-CAM), which is gradient-free localization mapping suitable for our analysis purposes.

We adopted Gildenblat's [32] CAM method library for score-CAM analysis. For the target layer, we chose the C2f module at the back of the Detection Model module from YOLO.

### Statistical analysis

For continuous variables in our study, non-parametric hypothesis tests were chosen due to non-normal distribution. For categorical variables, the *ꭕ*^2^ test was used.

## Results

### Dataset features

In total, 653 intra-oral photographs with 12,216 teeth and 8392 WSL bounding box annotations were included in our dataset. For the external testing subset, 1252 teeth (548 teeth presented with WSLs) and 842 WSLs were included. WSLs covered 14.8% of the area of the affected tooth crown (95% CI: 2.1–31.2%) in all photographs. With TW-YOLO, slices of intra-oral photographs were analyzed at their original resolution, and the median area for WSL bounding boxes was 2898 px (IQR: 1895–5080). The YOLOv5l model received downsized images, and the median area for WSLs decreased to 1135 px (IQR: 364–2423), significantly smaller than that in TW-YOLO. Judging by the standard from the COCO dataset [33], only four WSL bounding boxes had a small size (< 1024 pixels) during the prediction phase of the TW-YOLO model, whereas 395 had a small size when YOLOv5l was evaluated. It is obvious that the standard resize treatment for YOLOv5l not only decreased the size of all WSLs (*p* < 0.001) but also significantly increased the proportion of the small size bounding boxes (ꭕ^2^= 795, *p* < 0.001).

**Fig. 3:**
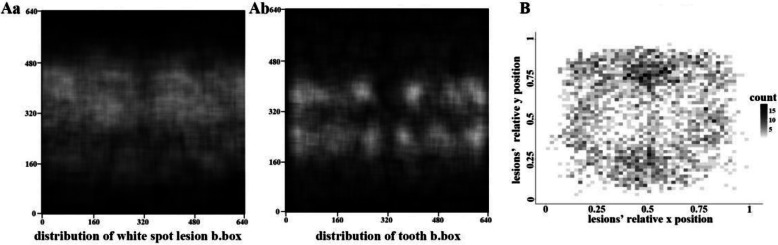
Analysis of dataset features. **A** Heatmap of locations of white spot lesion bounding boxes (**a**) and teeth (**b**). **B** Heatmap of the relative location of a white spot lesion to the tooth on which it was observed

Heatmaps of WSLs (Fig. 3Aa) and tooth bounding boxes (Fig. 3Ab) demonstrated their distribution in the central part of the photographs. For WSLs, the upper part was lighter than the lower part, suggesting that WSLs were primarily found in the upper jaw. The heatmap for the relative position of the WSLs to that of the corresponding tooth (Fig. 3B) showed that most WSLs were present on the peripheral area, especially on the upper and lower parts, which correspond to the peri-gingival area.

### Comparison of model performance

**Fig. 4:**
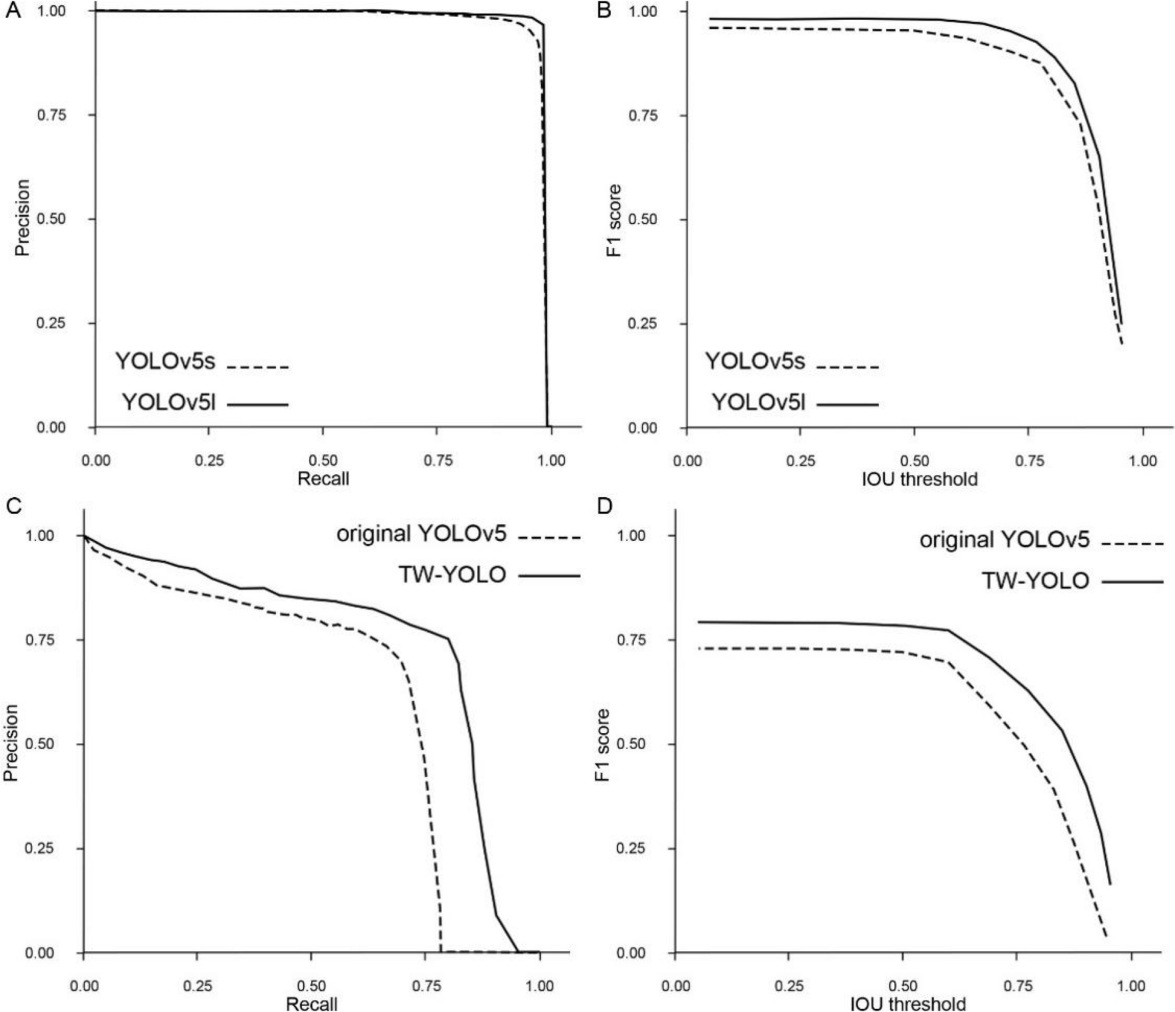
Comparison of performance metrics between different models. A Precision recall curve at 0.5 IOU threshold for teeth detection. B F1 score over IOU threshold curve for teeth detection. C Precision recall curve at 0.5 IOU threshold for white spot lesions. D F1 score over IOU threshold curve for white spot lesions

YOLOv5s is used for detecting tooth in the first half of our TW-YOLO model. It shares a similar architecture to the YOLOv5l model but is structurally simpler and contains fewer parameters. As our result demonstrated, for images resized to a 640 × 640 resolution, YOLOv5s performs rapid detection (8 ms per image). Despite its efficiency, it maintains high accuracy in tooth detction (Fig. 4A, B, Table 1), achieving a MaP of 0.95 at an IOU threshold of 0.5 (mAP@0.5) and a MaP of 0.73 across the full range of 0.5–0.95 IOU thresholds (mAP@0.5:0.95). The YOLOv5l model demonstrates superior accuracy compared with YOLOv5s, with an mAP@0.5 of 0.98 and an mAP@0.5:0.95 of 0.8, though it requires a longer inference time, at 12 ms per image. These results suggested that YOLOv5s has a lower computation time cost and comparable accuracy in tooth detection, thus suitable for tooth location detection as the first half of TW-YOLO model.

**Table 1:** Tooth detection accuracy for all models. Median values (95% confidence intervals) are shown and compared

Models	mAP@0.5	mAP@0.5:0.95	Time(ms)
YOLOv5s	0.95(0.93–0.97)	0.73(0.69–0.74)	8(6–25)
YOLOv5l	0.98(0.97–0.98)	0.80(0.80–0.81)	12(8–42)

*Abbreviations*: mAP@0.5: average precision at 0.5 intersection over union threshold; mAP@0.5:0.95: mean average precision over the range of intersection over union threshold from 0.5 to 0.95; Time: time needed for inference of a single image in micro-seconds

For WSLs, the detection accuracy for YOLOv5l and TW-YOLO at 0.5 IOU threshold and across 0.5 to 0.95 IOU threshold range was shown in the precision-recall curves (Fig. 4C) and F1-IOU curves (Fig. 4D)​. The pixel-wise Cohen's kappa coefficient was 0.76 for TW-YOLO and 0.62 for YOLOv5l. For secondary performance metrics (Table 2), when applying standard image resizing followed by YOLOv5l detection, the model achieved an mAP@0.5 of 0.69 and an mAP@0.5:0.95 of 0.45. However, when using TW-YOLO, the detection accuracy improved by approximately 10%, with an mAP@0.5 of 0.78 and an mAP@0.5:0.95 of 0.51. Among all 826 WSLs, TW-YOLO yielded 670 true positives (TPs), 156 false negatives (FNs), and 223 false positives (FPs). YOLOv5l, however, yielded 608 TPs, 218 FNs, and 252 FPs. The average inference time was 12 ms per image for YOLOv5l and 73 ms per image for TW-YOLO.

**Table 2:** White spot lesion detection accuracy for all models. Median values (95% confidence intervals) are shown and compared

Models	Kappa	mAP@0.5	mAP@0.5:0.95	TP	FN	FP	Time(ms)
YOLOv5l	0.62 (0.61–0.64)	0.69 (0.66–0.7)	0.45 (0.42–0.46)	608 (593–623)	218 (233–203)	252 (249–254)	12 (8–42)
TW-YOLO	0.76 (0.76–0.79)	0.78 (0.76–0.83)	0.51 (0.48–0.51)	670 (651–687)	156 (175–139)	223 (230–224)	73 (51–114)

*Abbreviations*: Kappa: pixelwise Cohen’s Kappa value between model prediction and ground truth annotation; mAP@0.5: average precision at 0.5 intersection over union threshold; mAP@0.5:0.95: mean average precision over the range of intersection over union threshold from 0.5 to 0.95; *TP* true positive, *FN* false negative, *FP* false positive, Time: time needed for inference of a single image in micro-seconds

### Explainability analysis

**Fig. 5:**
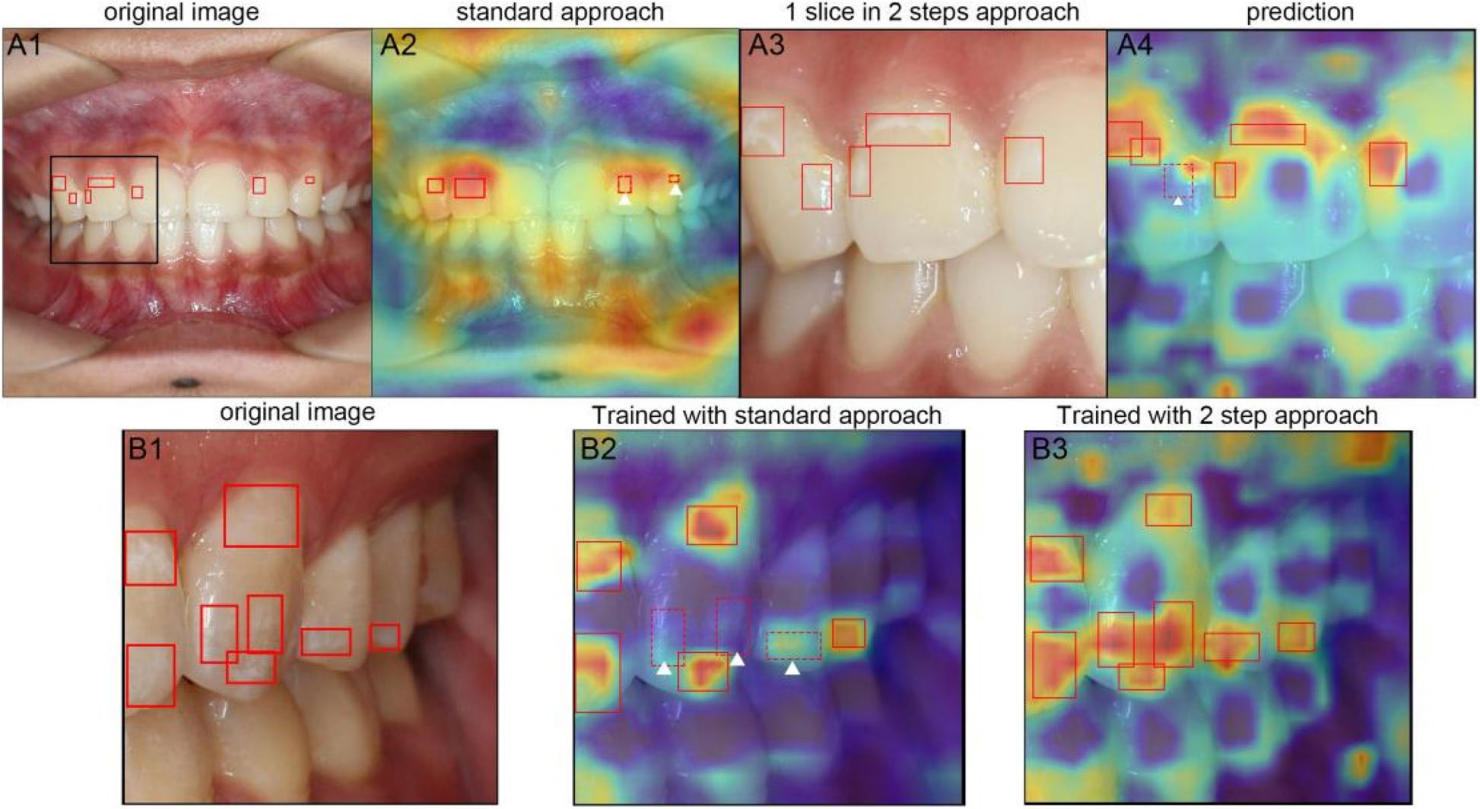
Explainability analysis of models. **A** Example image from our dataset (A1) and detection result, as well as overlayed score-CAM generated with YOLOv5l (A2). A regional crop of the intra-oral photograph (A3) and detection result, as well as overlayed score-CAM by TW-YOLO (A4), are also displayed to demonstrate differences in attention distribution between the models. **B** Intra-oral photograph (B1) along with detection and score-CAM overlay generated by YOLOv5l trained with the standard resize approach (B2). The detection result and score-CAM overlay generated by TW-YOLO are shown in B3

The score-CAM result for WSL detection (Fig. 5) demonstrated that while the YOLOv5l model mainly focused on teeth, considerable attention was diverted to the lips, oral mucosa, and cheek retractors when the standard resize approach was adopted (Fig. 5A2). In comparison, the TW-YOLO retained more detail within the images (Fig. 5A3, 4), and the model's attention was concentrated on the peripheral region of the tooth labial surface (Fig. 5A4), which coincided with the WSL distribution pattern (Fig. 3B). These features consequently resulted in more precise predictions.

The score-CAM results offered further insight into the superior performance of TW-YOLO when TW-YOLO image slices were compared with YOLOv5l image slices at the original resolution. For YOLOv5l (Fig. 5B2), the model’s attention was concentrated on the tip of the tooth and the peri-gingival area, leading to more FNs (Fig. 5B2). However, for TW-YOLO, attention was more evenly distributed along the peripheral area and more concentrated on salient features (Fig. 5B3).

## Discussion

Image data, as a central part of the patient record, play an essential role in the diagnostic and treatment workflow in orthodontic practice. The reading of these image data, however, still heavily depends on orthodontists’ manual work. This problem has become more evident as the amount of dental medical image data has grown exponentially. The development of artificial intelligence, especially in the field of deep learning, has introduced novel tools to increase dental image processing efficiency. Previous studies have shown that deep learning models performed well in cephalometric landmark detection [34] and dental implant systems classification [35]. These models could also detect and evaluate the severity of periodontitis [36] and periodontal bone loss [37].

In our study, we developed TW-YOLO, a novel network architecture specifically designed for detecting WSLs in intra-oral photographs. It demonstrated significantly stronger agreement with orthodontists' annotations than YOLOv5l, achieving an approximately 10% improvement in detection accuracy. TW-YOLO comprehensively outperformed YOLOv5l across all key metrics, including increased mAP@0.5, mAP@0.5:0.95, and TPs, as well as reduced FNs and FPs. Although inference time scaled Linearly to an average of 6× that of YOLOv5l due to its dual-network architecture, processing remained substantially faster than manual clinician annotation.

Several factors contributed to this superior performance. First, models adopted for fine-tuning were mainly pretrained with images with prominent subjects (target objects occupying > 10% of the image area). Thus, fine-tuning tends to yield superior results when the target objects in the dataset maintain relatively large proportions [10, 12, 38]. The approach developed by Askar et al. for WSL detection [8] involved cropping intra-oral photographs into slices of individual tooth size; this preserves the original resolution while reformulating detection into a classification task. Their method has achieved promising outcomes (R: 0.58–0.66; P: 0.67; AUC: 0.86). Nevertheless, the clinical utility of this approach remains limited by its reliance on labor-intensive manual cropping. By contrast, Özsunkar et al. directly downsized intra-oral images to 640 × 320 pixels before fine-tuning YOLOv5x, yielding suboptimal performance: a MaP of 0.454 at the 0.5 IOU threshold, detecting only 52% of WSLs and producing 133 TPs, 82 FNs, and 36 FPs [17]. As demonstrated in our study, simply downsizing intra-oral photographs significantly increases the proportion of small-size bounding boxes (0–1024 pixels), and detection of small-size targets is a well-recognized challenge in the object detection field [39, 40]. The suboptimal performance of the YOLO model observed in both the Özsunkar study and our study is primarily attributable to the increase in the number of small-size objects resulting from image downscaling.​​

Rich enamel textual information can be extracted from intra-oral photographs taken by a single-lens reflex camera. Multiple studies have shown that WSLs can be reliably assessed through meticulous, tooth-by-tooth examination of intra-oral photographs [3, 12]. This evidence aligns with our finding that tiled detection substantially improved accuracy by preserving critical details often lost after resolution reduction.

Compared with other generic tiling approaches that cut entire images into slices for sequential detection [19, 20], our method leverages the unique characteristics of intra-oral photographs by first detecting the location of all teeth and then performing slicing and detection in the cropped region. In this way, the area to be detected is reduced, saving detection time. Furthermore, from the perspective of score-CAM, the TW-YOLO model not only suppressed irrelevant areas (e.g. nostrils, lips, and gingival areas; Fig. 5) but also recognized salient features and focused on the distribution pattern of WSLs in our dataset. Applying this slicing windows strategy in both the training and prediction phases is more helpful than just applying it during the detection phase. As demonstrated in Fig. 5B, when sliced images at the original resolution are used as input, the YOLOv5 model can concentrate its attention on gingival margins and incisal edges of tooth surfaces; however, this approach results in the omission of many salient details, as most of the score-CAM overlay is still colored blue (Fig. 5B2). TW-YOLO, however, picked up more salient features (Fig. 5B3), thus enhancing its accuracy. The fact that the TW-YOLO network could implicitly learn the distribution pattern of WSLs is quite interesting and worth further investigation.

There are some limitations to our study. The size of our dataset was relatively small, and most photographs were taken after fixed appliances removal. With more image data, the fine-tuning of models could achieve better results. Additionally, the integration of an attention mechanism would allow for WSL distribution patterns to be explicitly taught to the models, which would be a more efficient approach than relying on the model to implicitly acquire this understanding over time. Finally, as this study employed a relatively early version of YOLO framework, its findings can’t fully represent performance of the recent updated models. Future research, leveraging novel frameworks integrating attention based techniques and Transformer technologies, is expected to significantly improve WSLs prediction accuracy.

## Conclusions

The novel TW-YOLO model not only demonstrated great accuracy but also showed near-perfect agreement with orthodontists' annotations. It enhanced the detection precision by effectively reducing the resolution degradation and concentrating on the key features of the tooth surface. Explainability analysis provided a better understanding of how these models perform in WSL detection and also indicated directions to explore for further improvements.

## Supplementary Information

Supplementary Material 1.

Supplementary Material 2.

Supplementary Material 3.

## Data Availability

The datasets used and analyzed during the current study are available from the corresponding author upon reasonable request.

## Abbreviations

YOLO: You Only Look Once Network
TW-YOLO: Tooth-to-WSL YOLO model
WSL: White spot lesion
mAP: Mean average precision
AP: Average precision
CAM: Class activation mapping
COCO: Common object in context
IOU: Intersection over union
Ms: Micro-seconds
