# Supplementary figures and images for: Tooth-to-white spot lesion YOLO: a novel model for white spot lesion detection
Source: BMC Oral Health. 2025 Oct 9;25:1577. doi: 10.1186/s12903-025-06936-w (PMC12512629; doi:10.1186/s12903-025-06936-w)

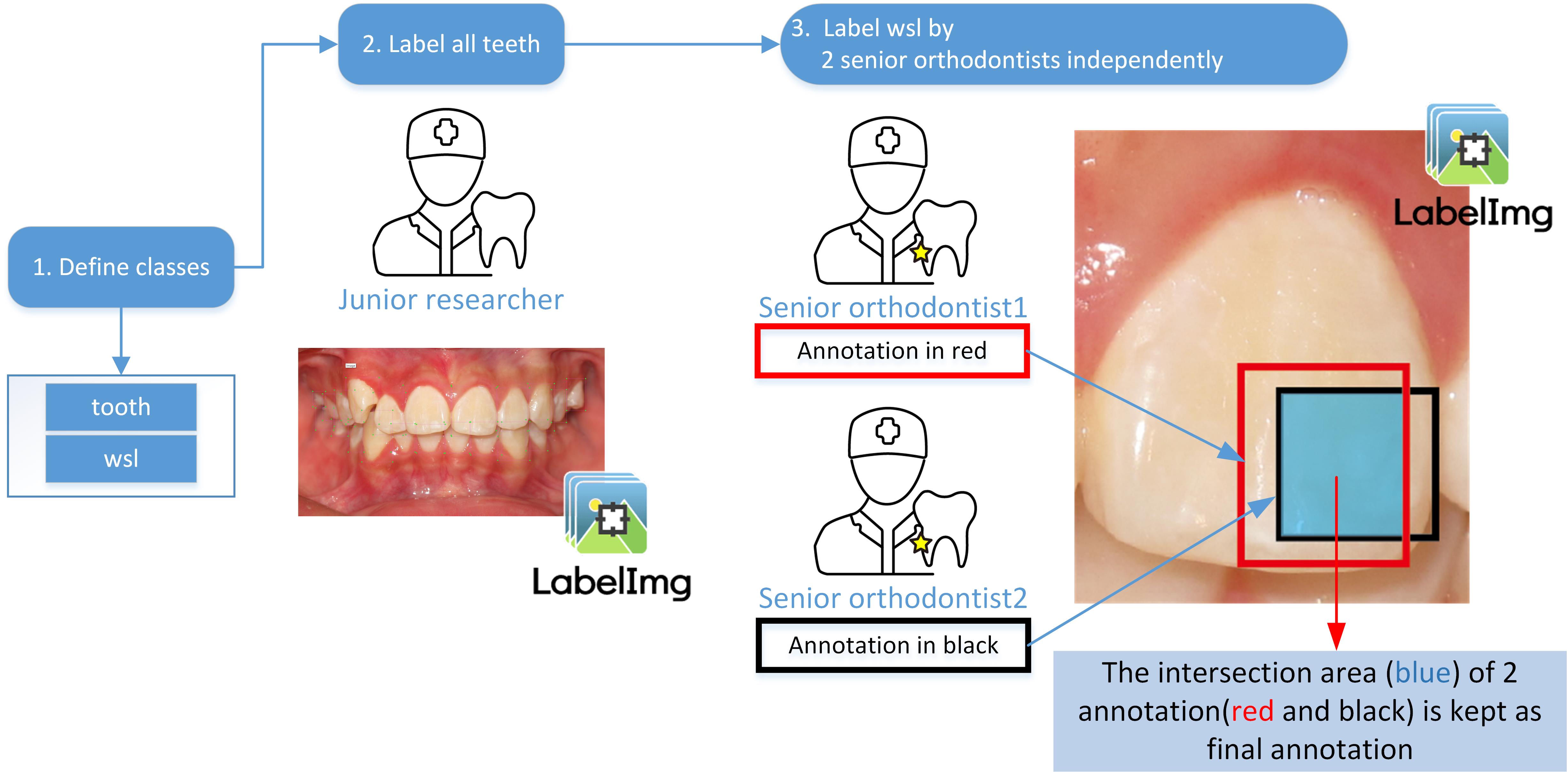

Supplement: Supplementary file 1 — Supplementary Material 1. [file 12903_2025_6936_MOESM1_ESM.jpg]
