# Supplementary material for: Tooth-to-white spot lesion YOLO: a novel model for white spot lesion detection
Source: BMC Oral Health. 2025 Oct 9;25:1577. doi: 10.1186/s12903-025-06936-w (PMC12512629; doi:10.1186/s12903-025-06936-w)
Supplement: Supplementary file 2 — Supplementary Material 2. [file 12903_2025_6936_MOESM2_ESM.docx]

# Checklist for Artificial Intelligence in Medical Imaging (CLAIM)

**TITLE or ABSTRACT**

**[x] 1 Identification as a study of AI methodology, specifying the category of technology used (eg, deep learning)**

We pointed out the current study is about developing a novel model for detection WSLs, and further clarified this in background and method section of abstract

**ABSTRACT**

**[x] 2 Structured summary of study design, methods, results, and conclusions**

The abstract is written in structured form according to journal’s requirement. A structured summary of study design, methods, results, and conclusions are all included.

**INTRODUCTION**

**[x] 3 Scientific and clinical background, including the intended use and clinical role of the AI approach**

We have discussed clinical background in the introduction section

**[x] 4 Study objectives and hypotheses**

The study objectives is stated as follow in the article: “Therefore, instead of simply downsizing an intra-oral photograph, a novel deep learning model is needed to fully utilize rich texture data in the original image. Inspired by the task partitioning paradigm[18] and the sliding windows strategy[19, 20], we developed a tooth-to-WSL You Only Look Once (TW-YOLO) model and compared its accuracy metrics with those of YOLO. The hypothesis to be tested is whether there are difference in accuracy between TW-YOLO and YOLO.”

**METHODS**

**[x] 5 Prospective or retrospective study**

The current study is a retrospective study. This is stated in the data collection section

**[x] 6 Study goal, such as model creation, exploratory study, feasibility study, noninferiority trial**

The current study is a model creation and evaluation study, as stated in the introduction

**[x] 7 Data sources**

As stated in the data collection section, “The current retrospective study acquired anonymized intra-oral photographs of orthodontic patients with WSLs from image archives in the Orthodontics Department of Foshan Stomatological Hospital, Foshan University. All intraoral frontal view photographs taken from January 1st, 2020, to October 31st, 2024, were included for further selection."

**[x] 8 Eligibility criteria: how, where, and when potentially eligible participants or studies were identified (eg, symptoms, results from previous tests, inclusion in registry, patient-care setting, location, dates)**

As stated in line 113-116: “Two senior orthodontic specialists assessed whether WSLs were present in the photograph, where WSLs were defined as ICDAS scores of 1 to 2. In case of disagreement, the decision was made by principal investigator. Only photographs with WSLs were included in the database."

**[x] 9 Data preprocessing steps**

As stated in line 146-148, “Data augmentation techniques, including random cropping and scaling, image rotation, and affine transformations, were employed to expand the effective dataset scale and enhance the spatial robustness of the deep learning model."

**[x] 10 Selection of data subsets, if applicable**

Not applicable

**[x] 11 Definitions of data elements, with references to common data elements**

As stated in line 130-134: “All image data were first annotated manually by two senior orthodontic specialists with LabelImg[23] by drawing bounding boxes around WSLs. The specialists first annotated 10 cases together, reaching a consensus, and then each specialist continued the annotation work independently. Finally, only regions selected by both orthodontic specialists were kept as annotations”

**[x] 12 De-identification methods**

As stated in line 116-118, “Patient de-identification was done by removing all meta data from image file, and covering the patient's nose and eyes in the photograph.”

**[x] 13 How missing data were handled**

Not applicable

**[x] 14 Definition of ground truth reference standard, in sufficient detail to allow replication**

The enamel decalcification index (EDI) was selected as ground truth reference standard. It is a classification and localization system commonly used in the evaluation of decalcification lesion in enamel.

**[x] 15 Rationale for choosing the reference standard (if alternatives exist)**

We mainly focus on studying whether deep learning model can reach consensus with orthodontic specialist, thus the annotation from specialist is set as ground truth.

**[x] 16 Source of ground truth annotations; qualifications and preparation of annotators**

As stated in line 130-134: “All image data were first annotated manually by two senior orthodontic specialists with LabelImg[23] by drawing bounding boxes around WSLs. The specialists first annotated 10 cases together, reaching a consensus, and then each specialist continued the annotation work independently. Finally, only regions selected by both orthodontic specialists were kept as annotations.”

**[x] 17 Annotation tools**

LabelImg software

**[x] 18 Measurement of inter- and intrarater variability; methods to mitigate variability and/or resolve discrepancies**

Intersection over union area is selected as inter- and intrarater variability measurement. As stated in line 130-134, we only kept the area selected by both specialist.

**[x] 19 Intended sample size and how it was determined**

As stated in line 149-154:” The minimum sample size was estimated based on the approach outlined in a previous study[26]. Based on a pilot study, we estimated that Cohen's kappa coefficient values were 0.6 and 0.77 for YOLOv5 and TW-YOLO, respectively. On average, WSLs covered 15% of the tooth enamel surfaces in the photographs. Given these parameters, at least 505 teeth with WSLs were needed. The external testing dataset included 548 teeth with WSLs, satisfying the minimum sample size requirement.”

**[x] 20 How data were assigned to partitions; specify proportions**

As stated in line 137-141: With a previous study[24] used as a guide, the 653 intra-oral photographs were randomly split into three groups (Fig 1): 457 images were used for training; 130, for validation; and 66, for hold-out testing. The images in the train-validation dataset (90% of all images) were used for model training and validation, and the images in the hold-out testing dataset (10% of all images) were used for performance evaluation.

**[x] Level at which partitions are disjoint (eg, image, study, patient, institution)**

Partitions are disjoint at image level

**[x] Detailed description of model, including inputs, outputs, all intermediate layers and connections**

Input for YOLO model is a batch of downsized images (640 by 640 pixels), and for TW-YOLO is a batch of original sized images. The architecture of our TW-YOLO model is stated in TW-YOLO model architecture section, in line 155-176. This novel model concatenate 2 YOLO model in tandem, and for specification of YOLOv5 model, including its output format, intermediate layers and connections can be obtained at its document page [1].

**[x] Software libraries, frameworks, and packages**

Pytorch framework (v2.8, on CUDA 12.6, python 3.12) was used for model finetuning and evaluation. Gildenblat's CAM library was used for explainability analysis.

**[x] Initialization of model parameters (eg, randomization, transfer learning)**

As stated in the article, we adopted transfer learning approach.

**[x] Details of training approach, including data augmentation, hyperparameters, number of models trained**

As stated in line 185-191: Fine-tuning of the YOLO network was performed by training models for 500 epochs with adaptive optimization hyperparameters. Specifically, batch size was set to 4 images per batch, learning rate initially was set to 0.01, final learning rate was set to 0.0001, Stochastic Gradient Descent (SGD) was used as optimizer. The loss function, comprising object detection loss (computed via intersection over union [IOU]) and classification loss (computed via Cross-Entropy), was evaluated in each epoch and backpropagated to update model weights.

**[x] Method of selecting the final model**

Model with best performance in both training and validation set is selected as final model.

**[x] Ensembling techniques, if applicable**

Not applicable

**[x] Metrics of model performance**

As stated in line 194-204: The primary evaluation metric was the pixel-wise Cohen’s kappa coefficient. We adopted this metric to evaluate the agreement between our orthodontists and the models regarding the boundaries of the WSLs. Other accuracy metrics included overall mean average precision (mAP@0.5:0.95), which was first introduced by the COCO detection challenge and has since become the most common evaluation metric for object detection accuracy. Average precision at the 0.5 IOU threshold (AP@0.5) and F1 score were chosen as secondary accuracy metrics. The evaluation metrics, including IOU, precision (P), recall (R), mAP@0.5:0.95, mAP@0.5, and F1 score, were calculated using methods outlined in prior research[8, 27]. The prediction time for each image was also tracked and compared.

**[x] Statistical measures of significance and uncertainty (eg, confidence intervals)**

Confidence intervals were calculated with bootstrap method and reported in table 1 and table 2

**[x] Robustness or sensitivity analysis**

Explainability analysis is performed with score-CAM, which is in essence sensitivity analysis.

**[x] Methods for explainability or interpretability (eg, saliency maps) and how they were validated**

Score-CAM is performed and saliency maps is drawn.

**[x] Validation or testing on external data**

This is stated in 137-141

**Results**

**[x] Flow of participants or cases, using a diagram to indicate inclusion and exclusion**

This is described in Figure 1.

**[x] Demographic and clinical characteristics of cases in each partition**

Data obtained in the current study is already de-indentificated, and demographic and clinical characteristics analysis is not applicable. But on the size and location of WSLs lesions, detailed information is given in line 232-251, and also in Figure 3.

**[x] Performance metrics for optimal model(s) on all data partitions**

This is reported in table 1, table 2 and the Comparison of model performance section.

**[x] Estimates of diagnostic accuracy and their precision (such as 95% confidence intervals)**

As stated in line 268-269: The pixel-wise Cohen's kappa coefficient was 0.76 for TW-YOLO and 0.62 for YOLOv5l. Which suggest that a near perfect agreement with orthodontic specialist is achieved by TW-YOLO, while sub-optimal agreement is achieved by YOLO model. 95% confidence interval is given in table 2.

**[x] Failure analysis of incorrectly classified cases**

This is explained in Explainability analysis section

**Discussion**

**[x] Study limitations, including potential bias, statistical uncertainty, and generalizability**

It is stated in line 363-372

**[x] Implications for practice, including the intended use and/or clinical role**

This is stated in line 305-314. Simply put, with application of deep learning model, the work load for assessing decalcification lesions on teeth can be alleviated.

**[ ] Registration number and name of registry**

The current study is a retrospective one. It is a pilot study aiming to develop a novel model, trying to find new ways to address detecting small WSLs on large intra-oral photographs. It did not fully met the standard of clinical trial, thus it is not registered.

**[x] Where the full study protocol can be accessed**

The study protocol, as well as datasets used can be accessed by contacting corresponding author.

**[x] Sources of funding and other support; role of funders**

Stated in line 403-406

Reference:

1. YOLOv5 document pages https://docs.ultralytics.com/zh/yolov5/tutorials/architecture_description/
