# Supplementary material for: Tooth-to-white spot lesion YOLO: a novel model for white spot lesion detection
Source: BMC Oral Health. 2025 Oct 9;25:1577. doi: 10.1186/s12903-025-06936-w (PMC12512629; doi:10.1186/s12903-025-06936-w)
Supplement: Supplementary file 3 — Supplementary Material 3. [file 12903_2025_6936_MOESM3_ESM.docx]

# Calculation algorithm for model performance metrics


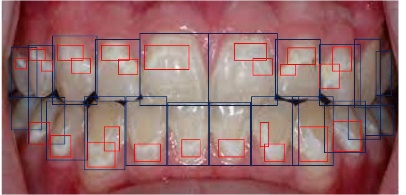
In the current study, an image was first annotated by orthodontists by drawing bounding boxes around objects and classifying them with predefined labels (teeth and white spot lesions), as indicated in Fig. 1.

Fig. 1 annotated image

A deep learning model will also predict bounding boxes for each class. Bounding boxes drawn by orthodontist are denoted as ground truths (g.t.), and those drawn by deep learning models are denoted as predictions (pred.).

## Intersection over union (IOU), precision, recall and F1 score .

g.t.

pred

intersection

union

Fig. illustration of intersection and union

1. **IOU:** For each ground truth bounding box of certain class, if there is a prediction bounding box for that class overlap with it, then IOU can be calculated as:

$$iou=\frac{intersection}{union}$$

Intersection and union in the formula are illustrated in Fig. 2.

1. **IOU threshold:** The minimal value for pred. box to be considered an overlap with g.t. box.
2. **TP, FP and FN:** For a group of g.t. boxes (n1) and a group of pred. boxes (n2), iou matrix of shape n1 x n2 can be calculated. For each g.t. box, there is 3 possible outcomes:
   1. A pred. box overlap with it (iou>=iou threshold), and they are the same class. This is a true positive (TP)
   2. A pred. box overlap with it (iou>=iou threshold), but they are not the same class. This is a false positive (FP)
   3. No pred. box overlap with it, this is a false negative (FN)
   4. If multiple pred. boxes are considered overlap with a g.t. box, and they are the same class, only the pred. box with biggest iou is considered TP, the rest are considered FP.
3. **Precision and recall:** precision is the proportion of correct prediction (TP) in all the pred. boxes (TP+FP) and recall is the proportion of correct prediction in all the g.t. boxes (TP+FN).

$$precision=\frac{TP}{TP+FP}$$

$$recall=\frac{TP}{TP+FN}$$

4. **Precision-recall curve:** The output of object detection model contains (1) shape and position information for bounding boxes, and (2) confidence score for each prediction. By sliding confidence score threshold from 0 to 1, more predictions will be included. In the current study, the range from 0 to 1 was spliced into 20 parts, precision and recall were calculated based on predictions that met corresponding confidence score threshold.
5. **F1 score:** F1 score is the harmonic means of precision and recall:

$$f1=\frac{precision\times recall\times2}{precsion+recall}$$

## Average precision

1. AP@0.5: IOU threshold was set at 0.5, precision recall curve was drawn, and the area under the curve was denoted as [AP@0.5](mailto:AP@0.5):

$$AP=\int_{r=0}^{1} p\left( r \right)d_{r}$$

2. mAP@0.5:0.95: The range between iou threshold 0.5 to 0.95 was divided into 10 parts, at each part AP is calculated, the average value is denoted as mAP@0.5:0.95

$$mAP=\frac{1}{10}\sum_{i=1}^{10} AP_{i}$$

## Pixel-wise Cohen’s Kappa

For each image, every pixel was treated as an observation, positive if included in bounding boxes and negative otherwise (Fig. 3). Cohen’s Kappa was calculated with “cohen_kappa_score” function from sklearn python package.


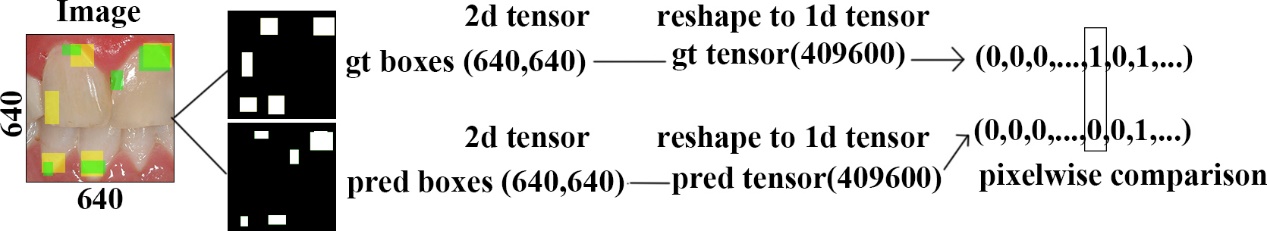


Fig. illustration of process for pixel-wise comparison
